# Influence of Fabrication Technique on Adhesion and Biofilm Formation of *Candida albicans* to Conventional, Milled, and 3D-Printed Denture Base Resin Materials: A Comparative In Vitro Study

**DOI:** 10.3390/polym15081836

**Published:** 2023-04-10

**Authors:** Reham B. Osman, Ghalia Khoder, Bahgat Fayed, Reena Arora Kedia, Yaser Elkareimi, Nawal Alharbi

**Affiliations:** 1 Department of Prosthodontics, Faculty of Dentistry, Cairo University, Giza 12613, Egypt; rehambosman@gmail.com; 2 Department of Pharmaceutics and Pharmaceutical Technology, College of Pharmacy, University of Sharjah, Sharjah P.O. Box 27272, United Arab Emirates; gkhoder@sharjah.ac.ae; 3 Research Institute for Medical and Health Sciences, University of Sharjah, Sharjah P.O. Box 27272, United Arab Emirates; bfayed@sharjah.ac.ae (B.F.); reenaarorakedia@gmail.com (R.A.K.); 4 Chemistry of Natural and Microbial Products Department, National Research Center, Giza 12622, Egypt; 5 Independent Researcher, Dubai, United Arab Emirates; yaser_y1177@yahoo.com; 6 Department of Prosthetic Dental Sciences, College of Dentistry, King Saud University, Riyadh 11451, Saudi Arabia

**Keywords:** 3D-printing, CAD/CAM milling, compression flask technique, manufacture technology, candida adhesion, candida biofilm formation, denture stomatitis

## Abstract

The aim of this study was to evaluate the adhesion and biofilm formation of *Candida albicans* (*C. albicans*) on conventionally fabricated, milled, and 3D-printed denture base resin materials in order to determine the susceptibility of denture contamination during clinical use. Specimens were incubated with *C. albicans* (ATCC 10231) for 1 and 24 h. Adhesion and biofilm formation of *C. albicans* were assessed using the field emission scanning electron microscopy (FESEM). The XTT (2,3-(2-methoxy-4-nitro-5-sulphophenyl)-5-[(phenylamino) carbonyl]-2H-tetrazolium hydroxide) assay was used for the quantification of fungal adhesion and biofilm formation. The data were analyzed using GraphPad Prism 8.02 for windows. One-way ANOVA with Tukey’s post hoc testing were performed with a statistical significance level set at α = 0.05. The quantitative XTT biofilm assay revealed significant differences in the biofilm formation of *C. albicans* between the three groups in the 24 h incubation period. The highest proportion of biofilm formation was observed in the 3D-printed group, followed by the conventional group, while the lowest candida biofilm formation was observed in the milled group. The difference in biofilm formation among the three tested dentures was statistically significant (*p* < 0.001). The manufacturing technique has an influence on the surface topography and microbiological properties of the fabricated denture base resin material. Additive 3D-printing technology results in increased candida adhesion and the roughest surface topography of maxillary resin denture base as compared to conventional flask compression and CAD/CAM milling techniques. In a clinical setting, patients wearing additively manufactured maxillary complete dentures are thus more susceptible to the development of candida-associated denture stomatitis and accordingly, strict oral hygiene measures and maintenance programs should be emphasized to patients.

## 1. Introduction

Complete denture and overdenture prostheses have long been used and are well-established treatment modalities for the rehabilitation of completely edentulous patients [1]. Conventionally, poly-methyl-methacrylate (PMMA)-based removable complete prostheses can be fabricated using compression and injection-mold or microwave-processed techniques [2,3]. PMMA-based materials are popularly used in removable prosthodontics because the material is easy to repair and light in weight, with a low cost. Following the breakthrough in digital dentistry and material development, namely in the field of prosthodontics, various clinical, laboratory procedures, and biomaterials have emerged for the fabrication of digital complete dentures [1,4]. Computer-aided design and computer-aided manufacture (CAD/CAM) of removable prostheses made from PMMA materials can be achieved either through additive/3D-printing or subtractive/milling manufacturing techniques [4,5,6,7]. Additive manufacturing of complete dentures is based on photo-polymerization of liquid resin, forming dentures on a layer-by-layer basis through a process known as 3D-printing. On the other hand, milled dentures are fabricated by milling of prefabricated, highly polymerized resin blanks.

CAD/CAM techniques, both milling and 3D-printed, offer several advantages over the conventional fabrication methods, such as shortened fabrication time, data archiving, and automated denture fabrication. The 3D-printing technique is a more sustainable approach for fabrication with less material waste, lower cost, and more potential for fabrication of detailed customized structures. Furthermore, opposite to milling, objects with a complex structure can be fabricated using 3D-printing as the tolerance of milling tools is not an issue [8].

The manufacturing technique utilized influences the surface topography of the material presented by surface defects, irregularities, cracks, and porosities [9]. Such defects provide a protective surface to which the microbes can bind and accordingly, the susceptibility of base material to the adhesion of microorganisms. The stepwise nature of the 3D-priniting technique results in the formation of what is known as stair-stepping phenomena, which is most apparent on curved surfaces [10,11]. Of much relevance is the palatal surface of maxillary complete dentures. Milled surfaces are characterized by parallel oriented lines created by milling burs [9]. Whereas, in the conventional fabrication technique, small, microscopic voids, porosities, and roughness are common, inherent to the conventional processing technique associated with linear and volumetric shrinkage of material [12,13].

The performance evaluation of differently fabricated resin base materials is no longer solely dependent on the physical and mechanical properties but also on the microbiological properties of the material. Candida-associated denture stomatitis is a well-known inflammatory condition of denture-bearing mucosa in edentulous patients wearing maxillary complete dentures, especially those with poor oral hygiene or those who are medically compromised and can cause both oral and systemic candidiasis [14]. Other denture-related factors that can lead to denture stomatitis are poor denture hygiene procedures and the roughness of resin base materials. Porosities and rough surfaces provide protective surfaces for irreversible adhesion of fungal cells and promote biofilm formation that act as a reservoir that represents the main source of infection [15,16]. Among various species available, *Candida albicans* (*C. albicans*) is the most commonly isolated species in cases of denture stomatitis [14]. Several studies compared the adhesion of *C. albicans* between conventional heat-polymerized and novel CAD/CAM additive or subtractive resin base materials [17,18,19]. However, in these studies, the specimens were either disc-shaped [17,18] or were highly polished, with a smooth surface finish [17,19], both of which are known factors that influence the microbial adhesion to the tested specimens [20].

Therefore, the aim of the current study was to simulate the actual clinical situation by evaluating the biofilm formation and comparing the adhesion of *C. albicans* to unpolished denture base resin specimens designed to simulate the curved part of the palatal cross-section of a maxillary complete denture fabricated with either the conventional flask compression technique or with CAD/CAM-milled or 3D-printed technology.

The null hypothesis was that there would be no difference in the candida adhesion and biofilm formation between conventionally fabricated, milled, and 3D-printed denture base resin materials.

## 2. Materials and Methods

### 2.1. Sample Preparation and Study Design

**Figure 1:**
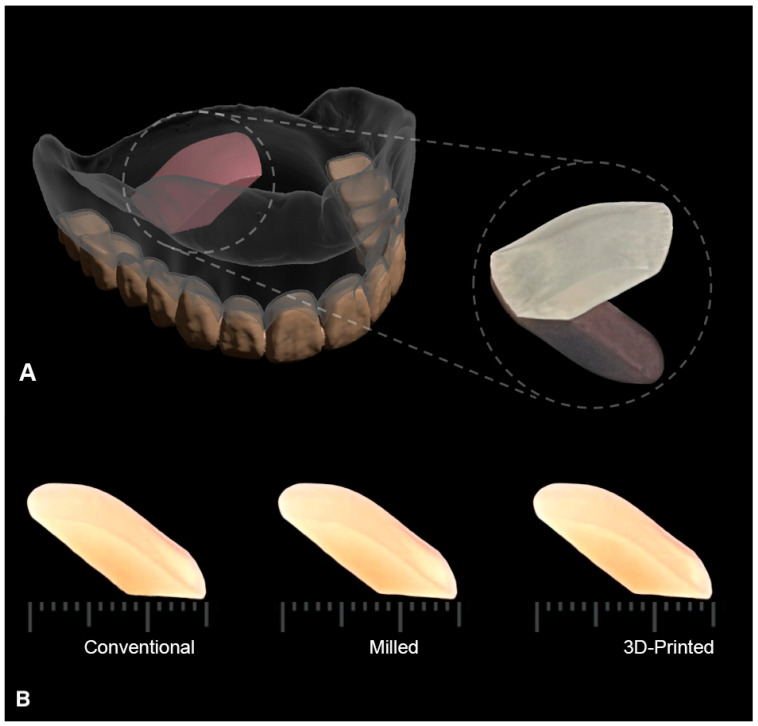
(**A**) Illustration of the designed test specimen as a section of the maxillary complete denture marked in pink. Enlarged specimen is shown on the right side. (**B**) Scale bar showing the standardized dimensions of the fabricated specimens in each test group: conventional, milled, and 3D-printed, to control all variables except for the material of fabrication.

The specimens were digitally designed to simulate the curved part of the palatal cross-section of a maxillary complete denture using Meshmixer open-source software (Autodesk, CA, USA) (Figure 1).

A total of 42 specimens were prepared and divided into three groups each (n = 14) based on the fabrication method. The fabrication techniques used were conventional compression molding (control), additive 3D-printing, and subtractive milling techniques. The biofilm formation of *C. albicans* was evaluated both qualitatively and quantitatively on the curved surface, simulating the fitting surface of the denture in all groups. The lower surface of specimens opposite to the test surface was designed to be almost flat to facilitate the examination of specimens under field emission scanning electron microscope (FESEM, Thermoscientific Apreon, Waltham, MA, USA) (Figure 1).

In each group, 9 specimens were used for fungal biofilm quantification using the XTT assay assessment in a 24 h period, 2 specimens were randomly selected for the qualitative FESEM examination after 1 and 24 h of incubation with *C. albicans*, and the other 3 specimens were used as control specimens without fungal incubation on the surface. The XTT assay of the 3 test groups (conventional, milled, and 3D-printed groups) was performed in triplicates in well plates. All the specimens were used as produced following the fabrication technique with no surface treatment being performed to any of the specimens’ surfaces.

#### 2.1.1. Conventional Compression Molding Technique

To standardize the specimens’ dimensions, the digital design file was 3D-printed in cast resin material (Fusia RF 080; DWS, Thiene, Italy) (n = 14) with a layer thickness of 0.05 mm following the manufacturer’s instructions. The printed cast material was then embedded in gypsum (Moldabaster S, Heraeus Kulzer GmBH, Hanau, Germany) to produce a two-piece mold using a copper flask (Varsttym Hanau; Buffalo, NY, USA). After the mold was created, the cast resin was eliminated using a clean brush and boiling water to which detergent had been added to eliminate all traces of the resin. The stone mold was then painted with an aqueous alginate-based separating medium (ISO-K; Candulor, Glattpark, Switzerland) and allowed to dry before the packing of the resin. The specimens were fabricated using a conventional compression-molding technique from heat-cured denture resin base material (major. base20; Moncalieri (TO), Italy) [3].

The denture resin was packed and cured using a long curing cycle at 90 °C with a pressure of 3000 psi for 3 h, followed by a holdout period at a temperature of 70 °C for 9 h. The holdout period allowed for the dissipation of the exothermic heat reaction within the processing flask to prevent any gaseous porosity before increasing the temperature for final processing. The cured specimens were then de-flasked by a flask ejector (Hanau; Louisville, KY, USA) after slow cooling at room temperature for 12 h to minimize the processing strains within the specimens. All specimens were stored in distilled water at room temperature until testing.

#### 2.1.2. CAD/CAM 3D-Printing Technique

**Figure 2:**
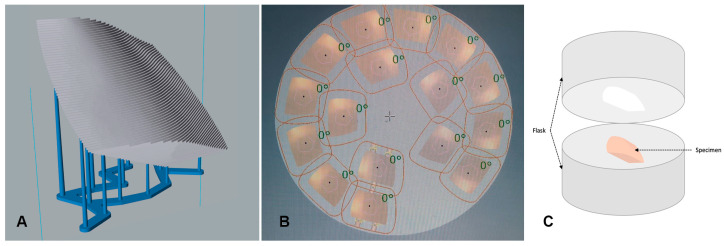
The manufacture of specimens in the (**A**) 3D-printed group, (**B**) milled group, and (**C**) conventional group. (**A**) The 3D-printed group, where the stair-stepping phenomena is evident on the fitting surface of the denture as a result of the manufacture layering process and the attachment of support structures to the opposite surface, representing the polished surface of the denture. (**B**) The milled specimens nested within the milling resin disc. (**C**) The conventional technique, where the specimen is invested for the flasking procedure.

Specimens were 3D-printed in NextDent Denture 3D+ material (NextDentTM, LOT: WY032NO1, shade: light pink) using an Asiga Digital Light Processing (DLP)-based 3D-printer (Asiga; Sydney, Australia). NextDent Denture 3D+ material is a biocompatible class IIa material with excellent mechanical properties, comparable to conventional resin base material, and is suitable for printing all types of removable denture bases. The specimens were printed using a 45° build angle, recommended for printing of maxillary complete dentures [21]. Figure 2A shows the attachment of support structures relative to printed specimens, and no support was attached to the surface representing the tissue-bearing area of dentures.

The printing layer thickness was 0.05 mm. After printing, all specimens were cleaned with 96% ethanol and post-cured using the UV-curing unit (Asiga Flash: Asiga; Sydney, Australia) following the manufacturer’s instructions.

#### 2.1.3. CAD/CAM Milling Technique

The standard tessellation language (STL) file of the designed specimens was exported into the milling machine and specimens were milled from pre-polymerized poly-methyl-methacrylate (PMMA) blocks (Opera Systems, Principaute de Monaco, Monaco, France). The design was virtually aligned within the disc with a 1.8 mm sprue thickness. Milling was performed using a 5-axis milling machine (Ceramill motion 2; Amann Girrbach AG, Koblach, Austria). Figure 2B shows the milled specimen nested within the resin disc.

### 2.2. Denture Microbial Susceptibility

To detect and measure fungal adhesion and biofilm formation, *Candida albicans* (ATCC 10231, Microbiologics; St. Cloud, MN, USA) from the American Type Culture Collection (ATCC) was used. This fungal strain was selected due to its ability to produce biofilm. Potato dextrose agar (Himedia, MH, Mumbai, India) was used to culture *C. albicans* aerobically at 37 °C. Sabouraud Dextrose Broth (SDB; Himedia, Mumbai, India) was used to form the *Candida albicans* biofilm.

#### 2.2.1. Microbial Culture and Biofilm Formation

**Figure 3:**
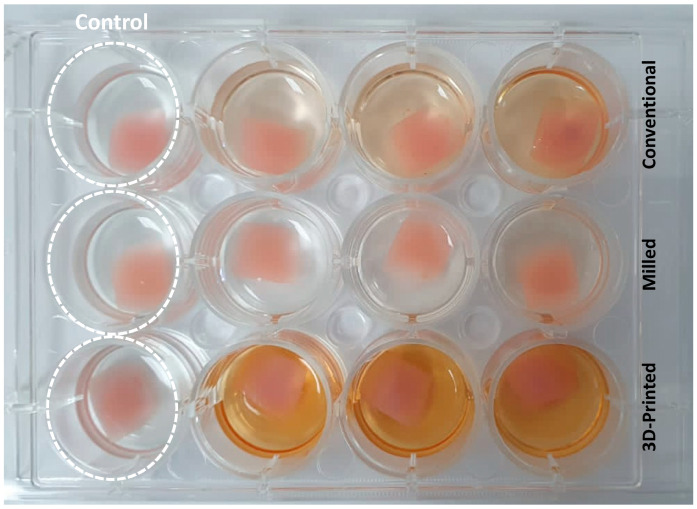
Biofilm quantification of the three test groups (conventional, milled, and 3D-printed groups) in well plates in triplicates after 24 h of incubation with *C. albicans* using the XTT assay. Control group represents samples from each group kept uninoculated with *C. albicans*.

*C. albicans* (ATCC 10231) was cultured in Sabouraud Dextrose Broth (SDB) and incubated at 37 °C for 24 h. SDB was prepared with 5% sucrose. The samples were sterilized by using 70% ethanol followed by exposure to UV light for 30 min, and afterwards, samples were placed at the bottom of a 12-well plate (Costar, Corning Incorporated, Tewksbury, MA, USA). Then, 1.5 mL of each microbial SDB broth (0.5 MCF, equivalent to 10^6^ microbial cells per mL) was added to each well of 12-well microtiter plates containing a denture sample of each tested group. The denture samples were kept incubated with candida culture aerobically for 1 h at 37 °C to evaluate the microbial adhesion by scanning electron microscope and for 24 h at 150 rpm using a shaking incubator (Labnet, Labnet International, Inc.; Mayfield Ave, Edison, NJ, USA) to evaluate the biofilm formation by the XTT assay. Uninoculated wells containing sterile SDB supplemented with 0.5% sucrose with the samples were considered as the negative controls [22,23]. All denture samples were placed in triplicates in 12-well microtiter plates (Figure 3).

#### 2.2.2. Biofilm Assessment by XTT Assay

Following incubation (1 and 24 h), the dentures were washed with phosphate buffer saline (PBS) to remove the non-adhered planktonic cells. Then, biofilm formation was measured by the XTT assay following the method used by Peeters et al., with minor modification to fit 12-well plate as follows: 5 mg/mL of XTT, and incubation between XTT and the cells was kept until the first sign of color changes [24].

Metabolic activity of the adhered cells was measured by the XTT (2,3-(2-methoxy-4-nitro-5-sulphophenyl)-5-[(phenylamino) carbonyl]-2H-tetrazolium hydroxide) (Sigma-Aldrich) reduction assay. Briefly, 1.5 mL of 5 mg/mL of XTT ((2,3-(2-methoxy-4-nitro-5-sulphophenyl)-5-[(phenylamino) carbonyl]-2H-tetrazolium hydroxide) (Abcam, Cambridge, UK) and 1 mM of menadione (Sigma-Aldrich, St. Louis, MI, USA) were added to each well. Following incubation in the dark at 37 °C, 150 rpm, 100 μL was transferred to 96-well plates once color changes started to appear. Absorbance of XTT-menadione was read at 490 nm using a microplate reader (LT-4500 Labtech, Pocklington, York, UK). The values were standardized per unit area of acrylic specimens (Abs/cm^2^), and the wells containing AS without candida cells were considered blanks. The assays were carried out in triplicate on 9 separate dentures and the average value was calculated [25].

#### 2.2.3. Qualitative Analysis of Microbial Adherence and Biofilm Formation Using Field Emission Scanning Electron Microscope (FESEM)

For the scanning electron microscope observation, denture samples were washed following incubation of 1 h (adhesion assay) or 24 h (biofilm assay) with 0.1M PBS one time. Denture samples were then air-dried in a desiccator, and finally sputter-coated with gold prior to observation. The 3D images were constructed using field emission scanning electron microscope (FESEM, Thermoscientific Apreo C, Waltham, MA, USA), available at the University of Sharjah, Advanced Materials Research Lab, M1, at 3000× magnification.

### 2.3. Statistical Analysis

Sample size calculation was based on similar previous studies, where 10 samples were selected to estimate a power of 0.85 at α = 0.05 [26]. The gathered data were tabulated and graphed using GraphPad Prism 8.02 for windows (GraphPad Inc., La Jolla, CA, USA). The normality of the data was checked using the Shapiro–Wilk test and found to be normally distributed. Bacterial absorbance and relative absorbance were analyzed by one-way analysis of variance (ANOVA) using Tukey’s multiple comparison test. The significance level was set at *α* = 0.05.

## 3. Results

**Figure 4:**
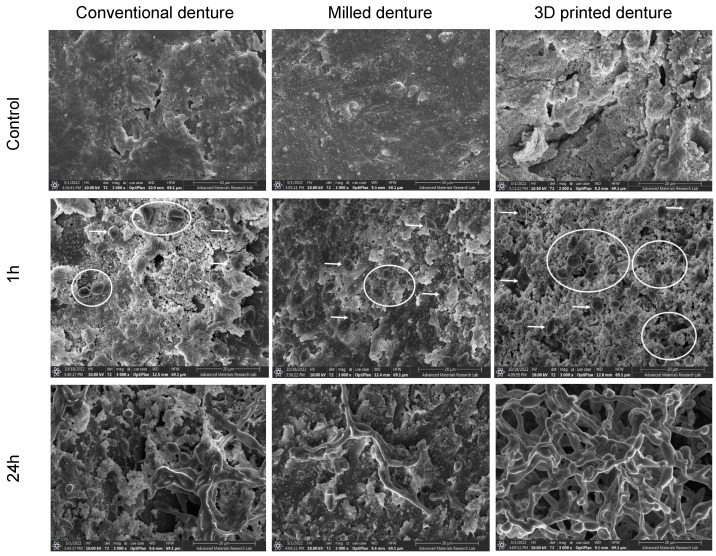
FESEM showing denture surfaces topographies (control) and *C. albicans* adherence (1 h) and biofilm growth (24 h) in all test specimens (magnification 3000×, scale bar: 20 um). White arrows show adherent separate ovoid fungal cells and white circles indicate clusters of cells presenting the initial stage of biofilm formation.

Optical analysis of FESEM revealed substantial differences in the appearance of specimens’ surfaces. Figure 4 shows the FESEM images of surfaces of both control and cultured specimens, and initial candida adhesion after a 1 h incubation period and biofilm formation of the *C. albicans* after 24 h of incubation. The 3D-printed dentures showed increased porosities of variable sizes, and deep grooves scattered all over the observed areas compared to the other two groups. Milled specimens showed the smoothest surface of all the groups with only grinding scratch lines detected on the surface of the specimens. The surface smoothness of the conventionally fabricated specimens was somewhere in between the other two groups, with some surface irregularities which were probably caused by stress relief and polymerization of resin during the fabrication process.

**Figure 5:**
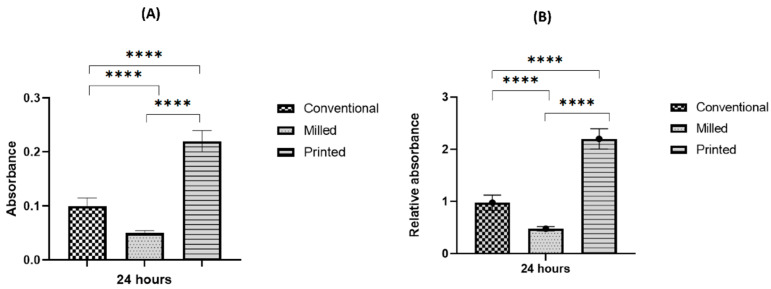
Biofilm assessment on conventional, milled, and 3D-printed groups. Average absorbance obtained with the XTT assay for *C. albicans* adhered for 24 h (**A**), and relative absorbance obtained with the XTT assay for *C. albicans* adhered for 24 h (**B**). Significance level is indicated by asterisks (**** *p* < 0.001). The data display the mean of either absorbance or relative absorbance.

**Figure 6:**
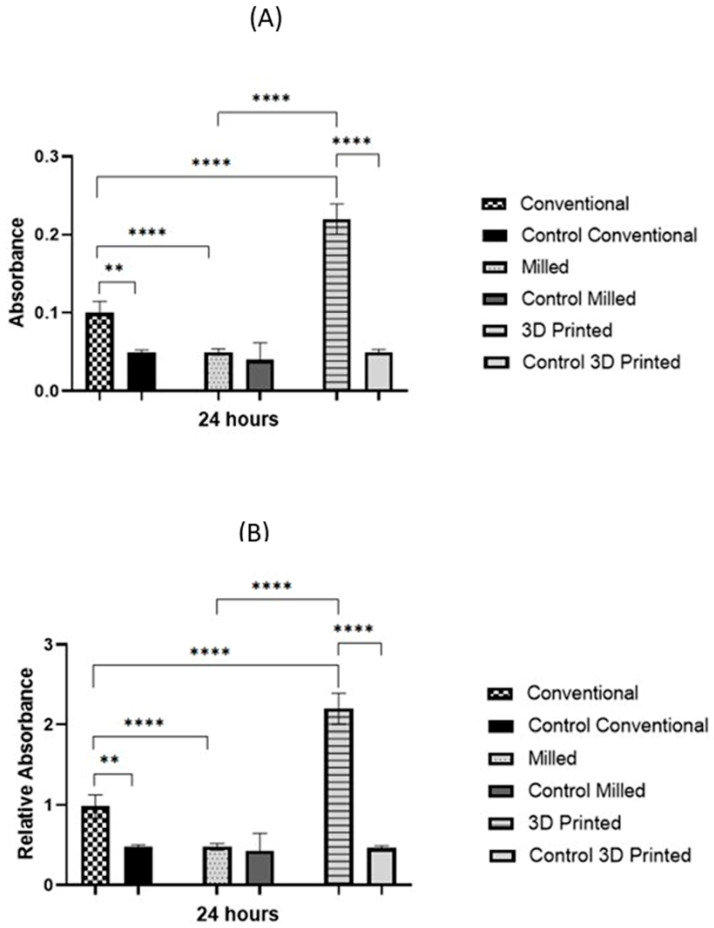
Biofilm assessment on conventional, milled, and 3D-printed groups and the control of each denture type. Average absorbance obtained with the XTT assay for *C. albicans* adhered for 24 h (**A**), and relative absorbance obtained with the XTT assay for *C. albicans* adhered for 24 h (**B**). Significance level is indicated by asterisks (** *p* = 0.005; ****, *p* < 0.001). The data display the mean of either absorbance or relative absorbance of each denture group to its control denture group.

At the 24 h incubation period, initially adherent *C. albicans* cells were clustered, forming typical hyphae that were more organized in the 3D-printed group compared to the other two groups (Figure 4), indicating a mature, well-developed formed biofilm. The quantitative XTT assay showed a significant difference in the biofilm formation between the groups in the 24 h incubation period (Figure 5). The relative absorbance measured by the XTT assay was used to reflect the biofilm formation on the tested dentures. In the conventional group, the relative absorbance was 0.98 ± 0.14, while in the milled and 3D-printed groups, the relative absorbance was 0.48 ± 0.14 and 2.2 ± 0.19, respectively. Milled specimens showed the lowest average fungal adhesion value, followed by conventional samples, and last was the 3D-printed group, with the highest adhesion values and the highest proportion of biofilm formation. The difference in biofilm formation among the three tested dentures was statistically significant (*p*-value < 0.001), as shown in Figure 6 (Table S1).

## 4. Discussion

Manufacturing techniques influence the surface properties of the manufactured part, which in turn impacts the microbiological properties of the material [27]. The purpose of this study was to evaluate the microbial response in terms of candida adhesion and biofilm formation on denture base resin materials manufactured using conventional compression molding technique, milling, and 3D-printing technologies. The null hypothesis was rejected as a significant difference in fungal adhesion and biofilm formation was found between the three groups in the 24 h incubation period. The candida adhesion and biofilm formation were significantly higher on 3D-printed specimens compared to heat-cured and milled CAD/CAM specimens. FESEM optical analysis revealed the roughest surface topography for unpolished 3D-printed specimens as compared with their milled counterparts, which exhibited the smoothest topography.

A number of authors found significant differences in fungal adhesion on polished and roughened denture base resin materials [28,29]. Thus, for the purpose of this study, it was deemed more clinically relevant that the specimens in all test groups be used as such when manufactured without performing any finishing or polishing procedures. In actual clinical settings, no finishing or polishing procedures are performed on the fitting surface of maxillary dentures so that not to alter the fit and retention of the fabricated denture.

The conversion degree of polymer material influences the amount of residual monomer in the processed material, and accordingly its physical, mechanical, and microbial properties [30,31]. Subtractive CAD/CAM discs are processed under high pressure and temperature, which reduces the level of residual monomer and enhances the structure of the processed material. In the current study, FESEM micrographs of the milled group revealed the smoothest surface topography (Figure 4), and the lowest levels of biofilm formation and candida adhesion of all the groups. Similar to our findings, Di Fiore et al. found that the milled PMMA samples had the lowest surface roughness before the polishing and the lowest microbial adhesion in the 90 min incubation period compared with heat-polymerized and additively manufactured samples [28].

In the printed specimens, the layers were linked stepwise, clearly demonstrating the stair-stepping phenomena (Figure 2A). The stepwise junction between the printing layers resulted in increased porosities and deep grooves that were observed in the surface structure. Such roughness is suggested to be the reason for the significantly increased candida adhesion that was observed in the 3D-printed group compared to the other two groups. Several authors suggested that surface roughness in the form of pits and fissures provides protective surfaces against shear forces and allows for irreversible adhesion of microorganisms [32,33,34,35]. Other authors attributed the increase in fungal adhesion on roughened surfaces to the increase in the surface area, which is provided by surface irregularities [29,36]. In the additive manufacturing technology, the quality and surface roughness of 3D-printed parts is dependent on the printing parameters selected (build angle, layer thickness, and orientation of support structures), the type of printer, and the underlying printing technology. All the above factors influence the degree of polymerization of the printed material, its physical properties, its surface roughness, and its microbiological properties [37,38,39,40,41]. Accordingly, to optimally simulate the actual clinical situation, a 45° (135°) [21] build angle with a 0.05 mm layer thickness was selected for the purpose of this study. Jin et al. demonstrated that such an angle results in the most accurate fitting surface of printed maxillary dentures [21]. Though Jin et al. referred to the angle used in their study as a 135° angle, it is equivalent to the 45° angle which was used in the current study considering the difference in the initial surface which was used to calculate the subsequent angles [21]. In their experimental setup, at the startup position, the support structure was attached to the fitting surface of the maxillary denture. Reduced layer thickness was selected to reduce the surface roughness of printed part, especially on curved geometries, though at the expense of an increased printing time [42].

Conflicting results are found in the literature when comparing the influence of different printing orientations (0°, 45°, 90°) on the physical and mechanical characteristics and the microbial response of 3D-printed denture base resin material. Shim et al. recommended a 90° build orientation because of its high accuracy, low roughness, and reduced attachment of *C. albicans*, disregarding its relatively low flexural strength [39]. On the contrary, Li et al. found no influence of the additive manufacturing method (SLA and DLP) and the print orientation (0°, 45°, 90°) on *C. albicans* adhesion on 3D-printed denture base resins [26]. However, the results of both studies should be regarded with caution as the use of disc-shaped specimens makes all their results clinically irrelevant and possibly different when considering the complex geometry of complete denture prostheses. Furthermore, from the authors’ point of view, when weighing printed dentures with improved strength and relatively increased microbial adhesion versus dentures with reduced mechanical properties and microbial adhesion, the preference could be in favor of the former. Printed dentures with reduced strength are more prone to repeated fractures, which might necessitate multiple maintenance events with its associated burden to both the dentists and patients alike. Dentures with increased microbial adhesion and improved strength could be better handled via emphasizing strict oral and denture hygiene measures. The weighed choice between mechanical, physical, and microbial properties of 3D-printed materials should thus be clinically driven based on the patient’s manual dexterity, compliance, and eating habits.

Arutyunov et al. found a difference in the adhesion index of candida on materials manufactured using the same manufacturing technology whether it was additive or subtractive denture base resin material. The slightest difference in the material composition alters its microbial characteristics [41,43]. Thus, for the sake of future studies, the difference in biofilm formation and candida adhesion between materials printed using different printers and different printable materials should be evaluated.

A direct comparison between our findings and previous studies that compared biofilm formation and candida adhesion on differently manufactured denture base resin materials was not possible and only general tendencies could be seen. In the current study, the specimens were designed to simulate the curved part of the palatal cross-section of the maxillary denture and a microbiological experiment was performed on the non-polished surface, representing the fitting surface of the denture as opposed to the flat-surface, polished disc-shaped specimens in the previous studies. The surface roughness and shape of the specimens were found to influence the degree of microorganisms’ adhesion on the examined part [20]. Consistent with our findings, Meirowitz et al. found the highest level of candida adhesion on the surface of additively manufactured specimens and the lowest with the subtractive ones, whereas the heat- and cold-polymerized specimens occupied an intermediate position between the other two groups [17]. Nevertheless, the authors correlated increased fungal adhesion on the printed surface to mucin adsorption rather than the surface roughness of the material. This could be attributed to the fact that the authors used polished, flat-surface specimens. On the contrary, Murat et al. did not perform polishing procedures and found a positive correlation between surface roughness and candida adhesion to milled CAD/CAM and conventional heat-polymerized resins, with statistically increased candida adhesion to conventional resins compared to CAD/CAM polymers [18]. In disagreement with all the previous findings, Schubert et al. found increased candida adhesion on CAD/CAM 3D-printed and milled oral splints compared to conventionally fabricated ones [19]. However, it must be highlighted that in their study, there was no specific mention of the selected printing parameters or specimen designs adopted, and the specimens were polished, resulting in a high gloss surface.

It is acknowledged that the oral cavity represents a very complex environment with the presence of saliva and the interaction of various microbial biofilms that may play a key role when considering the fungal adhesion to printed resin base materials. Clinical in vivo studies are strongly recommended to complement the findings of this in vitro study and enable better understanding of the microbial behavior of this novel introduced, printed denture base resin material.

## 5. Conclusions

The manufacturing technique had an influence on the surface topography and microbiological properties of the fabricated denture base resin material. Additive 3D-printing technology resulted in increased candida adhesion and the roughest surface topography of the maxillary resin denture base as compared to conventional flask compression and CAD/CAM milling techniques. In a clinical setting, patients wearing additively manufactured maxillary complete dentures are thus more susceptible to the development of candida-associated denture stomatitis, and accordingly, strict oral hygiene measures and maintenance programs should be emphasized to patients.

## Data Availability

Not applicable.

## Supplementary Materials

The following supporting information can be downloaded at: https://www.mdpi.com/article/10.3390/polym15081836/s1, Table S1: Average Optical Density (Avg OD) and Standard Deviation (STDEV).
